# Neutrophil-mediated type IV collagen degradation is elevated in patients with mild endoscopic ulcerative colitis reflecting early mucosal destruction

**DOI:** 10.1038/s41598-024-52208-y

**Published:** 2024-01-18

**Authors:** Marta S. Alexdottir, Martin Pehrsson, Viktor Domislovic, Line E. Godskesen, Aleksander Krag, Jens Kjeldsen, Marko Brinar, Ana Barisic, Anne-Christine Bay-Jensen, Morten A. Karsdal, Zeljko Krznaric, Joachim H. Mortensen

**Affiliations:** 1 Department of Biomarkers and Research, Nordic Bioscience, 2370 Herlev, Denmark; 2 Research Unit of Medical Gastroenterology, Department of Clinical Research, University of Southern Denmark, Odense, Denmark; 3 Department of Medical Gastrointestinal Diseases, Odense University Hospital, Odense, Denmark; 4 Department of Gastroenterology and Hepatology, University Hospital Center Zagreb, Zagreb, Croatia; 5 School of Medicine, University of Zagreb, Zagreb, Croatia

**Keywords:** Applied immunology, Biomarkers, Molecular medicine, Colonoscopy, Gastrointestinal diseases

## Abstract

Neutrophils play a significant role in sustaining chronic inflammation in Inflammatory Bowel Disease. The intestinal basement membrane acts as a barrier for immunological homeostasis, where the α3 and α4 chains of type IV collagen are expressed on the mucosal surface. We wanted to develop a biomarker reflecting early tissue injury, providing an opportunity for intervention. Two competitive enzyme-linked immunosorbent assays (ELISAs) quantifying human neutrophil elastase (HNE) degraded neo-epitopes of COL4A3 and COL4A4 were developed and investigated in two observational cohorts (n = 161, n = 100). A biomarker of MMP-mediated degradation of COL4A1 (C4M) was used for comparison. In Cohort 1, patients with mild endoscopic ulcerative colitis showed elevated levels of C4A3-HNE compared to those with severe disease. C4M had a strong positive correlation with disease activity. C4A3-HNE/C4M provided superior discrimination between mild and severe endoscopic disease and negatively correlated to disease activity. In Cohort 2, C4A4-HNE and C4A4-HNE/C4M showed similar trends. C4A3-HNE and C4A4-HNE possibly reflect early intestinal tissue injury. Combining the markers with a biomarker of another α-chain of the same collagen provides information on two distinct stages of mucosal damage. These biomarkers may be used to monitor disease flare-up in patients in remission, reducing the need for frequent endoscopic procedures.

## Introduction

Inflammatory bowel disease (IBD) is a chronic immune-mediated disease of the gastrointestinal (GI) tract, encompassed by Crohn’s disease (CD) and ulcerative colitis (UC). These two diseases differ by their location and depth of involvement, where UC involves inflammation limited to the colonic mucosa and submucosa, while CD causes transmural ulceration of any portion of the GI tract. IBD develops due to an abnormal immune response to the gut microbiota, where excessive inflammatory reactions lead to a continued deterioration of the intestinal barrier^1^. Comprising a thick mucus layer and a single layer of epithelial cells, the intestinal barrier provides both a physical and chemical barrier against invading microbes and harmful insults. Defects in this barrier lead to increased epithelial permeability, exposing the underlying tissue to luminal content and thus triggering an immunological response^2,3^. Impaired intestinal permeability is shown to precede clinical relapse in asymptomatic patients and can predict the course of the disease^4–6^. Therefore, improving the detection of subclinical inflammation could be crucial since early-stage IBD presents a unique window of opportunity for intervention, as the disease becomes a self-sustaining process once deep inflammation is established.

Neutrophils are the most abundant immune cells in circulation, the first responders of the innate immune system, and are best known for their rapid recruitment to sites of inflammation. Upon activation, neutrophils produce NETs (neutrophil extracellular traps), releasing proteases such as human neutrophil elastase (HNE) from granules stored in their cytoplasm^7^. Constant activation and excessive recruitment of neutrophils is a common feature in IBD, and their infiltration into the intestinal tissue has been correlated to disease activity^8^. HNE levels are elevated in plasma of IBD patients compared to healthy subjects, its expression is upregulated in the UC intestinal tissue, and HNE levels have been correlated with histopathological disease scores in CD^9–11^. As previously mentioned, disruption of the intestinal barrier is a hallmark of IBD as it leads to the exposure of microbes, furthering the initial inflammation. Therefore, the intestinal basement membrane (iBM) is of high importance for the structural integrity of the tissue. Acting as a front-line defense, the iBM is a collagen-rich matrix where type IV collagen is the major collagenous component^12^. Type IV collagen comprises six genetically distinct alpha chains, with three chains forming a singular collagen molecule. Three different α-chain compositions are known for type IV collagen, and they differ between tissues. The composition α1α1α2 is thought to be present throughout almost all basement membranes, and α5α5α6 is mainly localized in the epidermis. The third composition, α3α4α5, was only considered to be present in the kidney and alveoli of the lung (both organs require selective filtration through their BM)^13^. However, immunostainings of all six alpha chains in the human colon show that these chains are also present in the intestines, with α3 and α4 being solely expressed on the surface tip of the mucosa facing the lumen of the GI tract^14,15^.

Based on the expression pattern of α3 and α4, we hypothesized that these chains are readily accessible to initial inflammatory insults such as immune cell-secreted extracellular matrix (ECM)-degrading proteases. With neutrophils being recruited rapidly to novel sites of inflammation, the presence of HNE can also be linked to initial insult response. We therefore wanted to develop a biomarker reflecting early tissue injury, aiding in the implementation of prompt treatment. Here we present the development, technical validation, and biological evaluation of two competitive enzyme-linked immunosorbent assays (ELISAs) targeting HNE-generated neo-epitopes of type IV collagen α3 and α4, respectively. To our knowledge, this is the first time HNE-cleaved fragments of type IV collagen are used as biomarkers. Furthermore, we compared the novel biomarkers to another established type IV collagen biomarker (C4M) which reflects Matrix Metalloproteinase (MMP)-mediated degradation of the α1 chain.

## Materials and methods

### Assay development

#### Peptide identification through in-silico cleavage

Initial peptide identification was done through *in-silico* cleavage of the alpha-3 and alpha-4 chains of type IV collagen based on the known and very conserved cleavage sites of HNE. The cleavage sites are at the C-terminal of Ala, Val, and Ile, and based on in-house mass spec data, HNE also seems to cleave at the C-terminal of Serine. Cleavage fragments were generated manually and verified using a pre-made in-silico cleavage algorithm through the software Rapid Peptide Generator^16^ (RPG, Python v3.9). Fragments < 10 amino acids were avoided, as well as proline-glycine repeats. In-house MS databases were scanned to prevent the fragments of interest from being produced naturally by other proteases or as a passive byproduct. One neo-epitope fragment was chosen for HNE-degraded alpha-3 [EGTRPGPPGP] and another for HNE-degraded alpha-4 [TYPGRHGPPG].

#### Monoclonal antibody production, characterization of clones, and their specificity

Female Balb/C mice 6–7 weeks of age were immunized subcutaneously with 200 µl emulsified antigen and 100 µg of the C4A3-HNE [EGTRPGPPGP-GGC-KLH] or C4A4-HNE [TYPGRHGPPG-GGC-KLH] neo-epitope peptide. Immunizations were performed consecutively at 2-week intervals until stable sera titer levels were reached. The mouse with the highest titer was selected for fusion and rested for one month. The mouse was subsequently boosted intravenously with 50 µg of the respective immunogenic peptide in 100 µl of 0.9% NaCl solution three days before isolation of the spleen for cell fusion. Hybridoma cells were produced by fusion of the mouse spleen cells with SP2/0 myeloma cells, as described by Grefter et al.^17^. The hybridoma cells were cloned using a semi-solid medium method and transferred into 96-well microtiter plates for further growth, where the limiting dilution method was applied to promote monoclonal growth. All methods were carried out in accordance with relevant guidelines and regulations, and reported in accordance with the ARRIVE guidelines. Supernatants were screened for reactivity to the respective neo-epitopes using an indirect ELISA with streptavidin-coated microtiter plates and biotinylated peptides for C4A3-HNE [EGTRPGPPGP-K-Biotin] or C4A4-HNE [TYPGRHGPPG-K-Biotin]. Specificities of the clones to the free peptides [EGTRPGPPGP or TYPGRHGPPG], elongated peptides [SEGTRPGPPGP or VTYPGRHGPPG], truncated peptides [_GTRPGPPGP or _YPGRHGPPG] and nonsense peptides were tested. Cross-reactivity between C4A3-HNE and C4A4 was also tested. The supernatants were purified using HiTrap affinity columns (GE Healthcare Life Science, Little Chalfont, Buckinghamshire, UK) according to the manufacturer’s instructions. Antibodies were then further labeled with activated peroxidase from horse radish with a Peroxidase Labeling Kit, according to the kit protocol (Roche Diagnostics, Mannheim, Germany, CAT no. 11829696001).

#### ELISA protocol

Preliminary experiments were carried out where the assay reagents, their concentrations, and incubation periods were optimized. The final competitive ELISA procedures are as follows: A 96-well streptavidin plate was coated with synthetic biotinylated peptide (100 µl/well) diluted in assay buffer (25 mM PBS-BTB 6 g NaCl/L, pH 7.4 for C4A3-HNE and 25 mM PBS-BTB 8 g NaCl/L, pH 7.4 for C4A4-HNE), and incubated for 30 min at 20 °C in the dark while shaking at 300 rpm. After each incubation step, the plates were washed with washing buffer (25 mM Tris, 50 mM NaCl, 0.1% (v/v) Tween-20, pH 7.2) using a standardized ELISA plate washer (BioTek Instruments, Microplate washer, Elx405 Select CW). Standards and serum samples (20 µl/well) were diluted appropriately and added to the plate, followed by the addition of the horse-radish peroxidase-conjugated monoclonal antibody (100 µl/well) diluted in assay buffer and incubated for 1 h at 20 °C in the dark while shaking at 300 rpm. Finally, BM Chemiluminescence ELISA Substrate (Merck, CAT no. 11582950001) was added to each well and incubated for 3 min. The plates were then read using a fluorescent plate reader (Fluroskan FL, Thermo Fisher), with a read light emission at 1.000 ms and no filter. Standard curves were plotted using a four-parametric mathematical fit model. The C4M biomarker (α-1 chain of type IV collagen) was also measured using ELISA. An exact protocol can be found in the technical paper by Sand et al.^18^.

### Technical evaluation

#### Proof-of-concept cleavage

Intial cleavage experiments included incubating active active neutrophil elastase purified from human cells by SDS-page (Abcam, cat. no. ab280938) with native type IV collagen (Sigma-Aldrich, cat no. CC076). However, due to the complex structure of collagen molecules, their poor solubility, and their tendency to assemble into fibrillar structures, synthetic peptides mimicking the type IV collagen alpha-3 and -4 chains were eventually designed, with the target sequence in the middle (Genscript). The neo-epitopes were then generated in vitro by incubating active HNE (Abcam, cat. no. ab280938) with the synthetic peptides in a 100 mM Tris buffer with 500 mM NaCl (pH 7.5). To demonstrate that the neo-epitope fragments could not be generated by the same proteases as C4M, the synthetic peptides were incubated with MMP-2 (Merck, cat. no. PF037), MMP-9 (Merck, cat. no. PF038) and MMP-12 (RnD systems, cat. no. 917-MPB) in a 50 mM Tris buffer with 200 mM NaCl, 10 mM CaCl2, and 100 µM ZnCl (pH 7.5). All samples were incubated for 4 h at 37 °C in a reaction volume of 200 µl. MMPs were activated with 4-aminophenylmercuric acetate (APMA) before use (MMP-9 and -12 overnight, and MMP-2 for 2 h). The protein-to-protease ratio was 100:1. The proteolytic reaction of HNE was inhibited by adding the serine protease inhibitor AEBSF to a final concentration of 1 mM, while MMPs were inhibited using EDTA at a final concentration of 25 µM. Samples containing only uncleaved peptides or cleavage buffer served as experimental controls. Cleavage experiments were carried out in two biological replicates, with two technical replicates within each experiment.

#### Technical validation

Technical validation of the assays was performed to evaluate robustness, accuracy, precision, interference, and stability. The lower limit of blank (LLOB) was determined by measuring 60 assay buffer replicates. LLOB is defined as  ± 3xSD of buffer (blank) with no acceptance criterion. The lower limit of quantification (LLOQ) is defined as the lowest analyte concentration in serum, where the CV% of the precision profile equals 25%. LLOQ was determined by measuring four low-level human serum samples in three replicates in five separate runs. The upper limit of quantification (ULOQ) is defined as the highest standard point of the assay’s standard curve with acceptable accuracy and precision. ULOQ was determined by ten independent runs of the standard curve. The standard curve robustness and the half-maximal inhibition concentration (IC50) were also determined. The analytical measurement range is defined as the range between LLOQ and ULOQ. To assess linearity, four human serum samples were diluted from two-fold down to an eight-fold dilution. The percentage recovery from the undiluted sample was calculated with an acceptance criterion of 100%  ± 25. The reportable measurement range is based on the accepted dilution recovery and is defined as the range between LLOQ and ULOQ corrected for the maximum validated dilution. The inter- and intra-assay variations were determined by ten independent runs of eight human serum samples covering the quantifiable measurement range of the assays and two kit controls, all in double determinations. The acceptance criterion for inter-intra variability was a CV% < 15. To evaluate the accuracy of the assay and any potential matrix effects, matrix–matrix spiking recovery was determined, where three sets of serum samples with high and low analyte concentrations were spiked into each other in different proportions. The recovery percentage was calculated based on expected and measured concentrations, with a RE% criterion of  ± 25. Analyte stability in serum was assessed by exposing three human serum samples to four freeze and thaw cycles. The percentage recovery was calculated using the respective uncycled serum sample as a reference. To test whether common substances found in blood interfered with the assay analytes, an interference panel of biotin (0–100 ng/ml), hemoglobin (low = 2.5 mg/ml, high = 5 mg/ml) and lipid (low = 1.5 mg/ml, high = 5 mg/ml) was spiked into three individual human serum samples. The recovery percentage of the analyte for each interference sample was calculated with the respective control sample as a reference, with an acceptance criterion of 100% ± 20.

### Clinical evaluation

#### Patient samples

Two patient cohorts were used for the study at hand. The clinical evaluation of the biomarkers focused solely on patients with ulcerative colitis. Cohort 1 was obtained from Odense University Hospital, Denmark. Study subjects were enrolled in a prospective observational study (ClinicalTrials.gov ID: NCT02612103), including patients with CD (n = 60) and UC (n = 101). Before serum sample collection, informed signed consent was collected from each study subject. At the time of sampling, data on standard laboratory parameters, Montreal classifications, and disease activity was collected for all patients. The study was approved by the Regional Ethics Committee of Southern Denmark (journal number: S-20150107) and conducted according to the Declaration of Helsinki. To assess endoscopic disease activity, The Mayo endoscopic subscore was applied for UC (0: inactive disease; 1: mild activity, 2: moderate activity, 3: severe activity). Cohort 2 was obtained from the Clinical Hospital Centre Zagreb (Department of Gastroenterology and Hepatology) and included patients with CD (n = 72) and UC (n = 28). Serum samples were collected after informed signed patient consent. The study was approved by the University Hospital Center Zagreb Ethical Board (approval no. 02/21AG) and conducted according to the Declaration of Helsinki. Patients with co-morbidities and extra-intestinal manifestations were excluded from the study. Demographical data, disease activity, and treatment history were obtained from electronic medical records and questionnaires, while anthropometric parameters were measured upon inclusion. Endoscopic disease activity for UC was based on the Mayo Endoscopic score, where endoscopy was performed within three months of blood sampling. To limit the potential impact of this delay, the cohort only included patients who were without intervention in the time between blood sampling and endoscopy. The endoscopic scores were prospectively validated based on routine endoscopy and scoring by an experienced IBD endoscopist. Serum samples from 67 healthy donors (HDs) were obtained from BioIVT, not matched for age and gender (Westbury, NY, USA). Preliminary biological relevance was assessed in commercial serum samples obtained from patients with CD and UC (ProteoGenex, Inglewood, CA, USA). All research was performed in accordance with relevant guidelines/regulations.

### Statistical analysis

All data were considered non-parametric after a visual assessment of normality using density plots. Differences between patient groups were compared using Mann–Whitney *U*-tests (for two groups) or Kruskal–Wallis with Dunn’s test, Bonferroni-corrected for multiple comparisons (for multiple groups). Spearman’s ranked correlation was applied to assess the relationship between biomarkers, disease scoring, laboratory parameters, and common demographic variables. Receiver operating characteristics (ROC) statistics with the area under the curve (AUC) as an overall measure of fit were used to assess the discriminative capacity of the biomarkers. Sensitivity and specificity metrics were obtained by determining the optimal cut-off using Youden’s J statistic. AUC values were presented with their corresponding 95% confidence intervals (CI). A linear regression model was applied to assess whether demographical variables affected the biomarker levels, correcting for age, gender, and BMI. The model fit was evaluated through residual analysis and qq-plots. Univariate (method: enter, data not shown) and multivariate logistic regression analysis was applied to identify whether demographic variables were significantly associated with the odds of having severe endoscopic disease, using mild (0) vs. severe (1) as a binary variable. Data analysis and visualization were performed using Rstudio (version 4.1.2; Rstudio, Boston, MA, United States) and GraphPad Prism 9.2.0 (GraphPad Software, San Diego, CA, United States). *P*-values < 0.05 were considered statistically significant.

## Results

### C4A3-HNE and C4A4-HNE are highly specific ELISA assays

**Figure 1:**
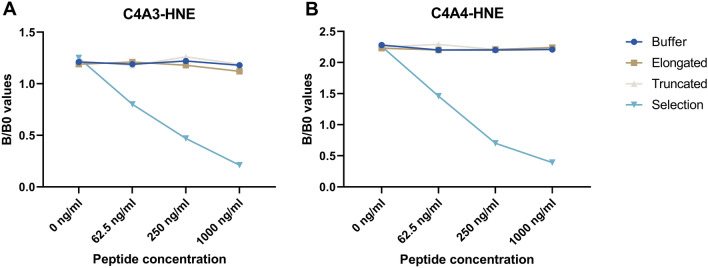
C4A3-HNE and C4A4-HNE monoclonal antibodies are highly specific towards their respective neo-epitopes. Dose-dependent reactivity was seen for both monoclonal antibodies when incubated with the selection peptides, mimicking the respective neo-epitopes they were raised against. B/B0: Sample signal/blank signal.

The specificity of the monoclonal antibodies raised against the neo-epitopes C4A3-HNE (Fig. 1A) and C4A4-HNE (Fig. 1B) was evaluated. For both assays, the selection peptide representing the neo-epitope inhibited the signal in a dose-dependent manner. When a mismatch was introduced by using the elongated and truncated peptides, the signal inhibition was on par with the blank (no reactivity).

### HNE releases the neo-epitopes C4A3-HNE and C4A4-HNE in vitro

**Figure 2:**
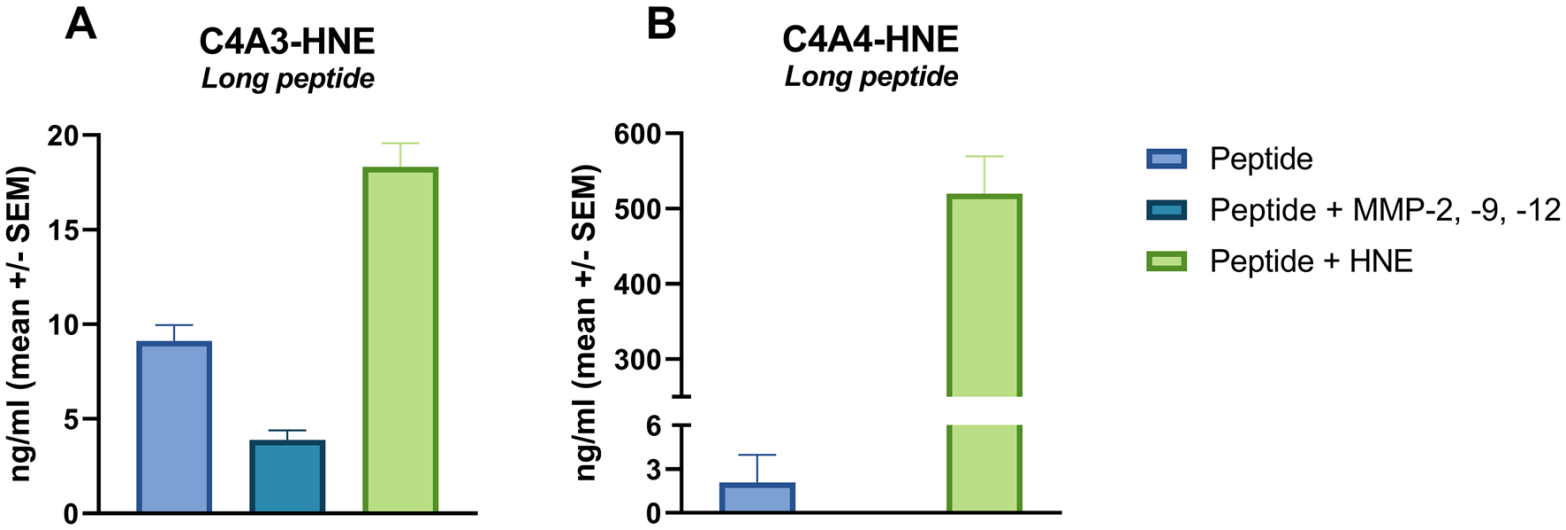
C4A3 and C4A4 neo-epitopes are released upon HNE cleavage, not MMP cleavage. Synthetic peptides mimicking type IV collagen alpha-3 (**A**) and alpha-4 (**B**) chains were incubated with active HNE or MMPs for 4 h. Peptide (without protease) was used as a negative control. HNE released the neo-epitope upon incubation with the peptides. Small amounts of C4A3-HNE were observed in the undigested sample (peptide) and the sample digested by MMPs. Likewise, a low amount of C4A4-HNE was observed in the undigested sample (**A** and **B**). Buffer was used as a background control; shown values are background subtracted.

We investigated the ability of HNE to degrade and release the neo-epitopes in vitro by incubating synthetic peptides mimicking the type IV collagen alpha-3 and -4 chains with active HNE. The peptides were also incubated with MMP-2, -9, and -12 to demonstrate that MMPs could not generate the neo-epitopes. The results showed that HNE could generate both neo-epitopes, as measured by their respective assays. Small amounts of C4A3-HNE were observed in the undigested sample (peptide) and the sample digested by MMPs. Likewise, a low amount of C4A4-HNE was observed in the undigested sample (Fig. 2A,B). Additionally, native type IV collagen was incubated with active HNE, where HNE was not able to release the neo-epitopes (Supplementary Figure S1).

### C4A3-HNE and C4A4-HNE are technically robust ELISA assays

The technical performance of the C4A3-HNE and C4A4-HNE assays in serum was further assessed through different technical validation steps, summarized in Supplementary Table S1. Intra- and inter-assay precision was determined to be  < 15% for both assays, passing the acceptance criterion. Accuracy (%RE) was determined to be between  − 4%–22% for C4A3 and  − 2.7–14.7% for C4A4. The linearity study showed acceptable dilution recovery of up to 25% dilution for both assays. Based on these data, the maximum validated dilution of human serum samples is a dilution of 1 + 3. Both analytes were stable for up to five freeze/thaw cycles in human serum. No interference was detected from low or high contents of biotin, lipemia, or hemoglobin in serum. These results indicate that C4A3-HNE and C4A4-HNE are technically robust assays and can be used for testing human serum samples.

### C4A3-HNE and C4A4-HNE are elevated in IBD

**Figure 3:**
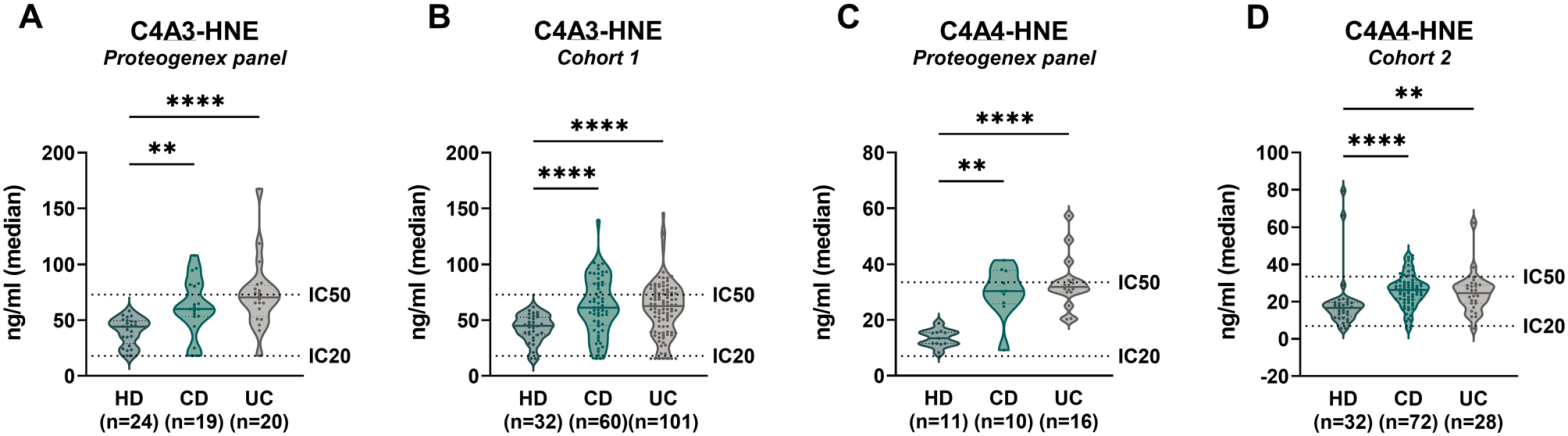
C4A3-HNE and C4A4-HNE are significantly elevated in patients with IBD compared to healthy donors. (**A**) C4A3-HNE levels are elevated in patients with CD and UC compared to healthy donors in commercial samples (*p* < 0.01 and  < 0.0001) and in Cohort 1 (**B**) (both *p* < 0.0001). (**C**) C4A4-HNE levels are elevated in patients with CD and UC compared to healthy donors in commercial samples (*p* < 0.01 and  < 0.0001) and in Cohort 2 (**D**) (*p* < 0.0001 and *p* < 0.01). Data is presented with violin plots, depicting the distribution and density of the data points with the horizontal line representing the median. Differences between groups were analysed using Dunn’s test with Bonferroni correction for MCP. **p < 0.01; ****p < 0.0001.

Biological relevance was assessed in IBD samples and healthy donors. C4A3-HNE was elevated in patients with CD and UC compared to healthy donors (HD vs. CD vs. UC [IQR]: 44.12 ng/ml [29.01, 49.50] vs. 60.00 ng/ml [53.31, 81.86] vs. 70.43 ng/ml [53.27, 81.21], *p* < 0.01 and  < 0.0001). The results were validated in Cohort 1 (HD vs. CD vs. UC [IQR]: 45.01 ng/ml [36.98, 52.80] vs. 61.24 ng/ml [45.21, 82.49] vs. 62.75 ng/ml [39.55, 75.20], both *p < *0.0001) (Fig. 3A,B). C4A4-HNE was elevated in patients with CD and UC compared to healthy donors (HD vs. CD vs. UC [IQR]: 13.45 ng/ml [11.54, 15.54] vs. 30.35 ng/ml [25.85, 37.80] vs. 31.85 ng/ml [29.98, 34.41], *p* < 0.01 and  < 0.0001). The results were validated in Cohort 2 (HD vs. CD vs. UC [IQR]: 16.68 ng/ml [11.93, 19.55] vs. 26.33 ng/ml [21.05, 30.30] vs. 24.66 ng/ml [19.24, 28.93], *p < *0.0001 and  < 0.01) (Fig. 3C,D). The discriminative capabilities of the biomarkers can be seen in Supplementary Table S2.

### Cohort characteristics

The clinical evaluation of the biomarkers focused solely on patients with ulcerative colitis.

#### Cohort 1

The median patient age was 45 years [IQR: 34, 57], with an even gender distribution (42% female vs. 58% male). Only 11% of patients (n = 12) were on no form of treatment. The majority of patients (50%, n = 54) were in remission according to the partial Mayo score, while 4% (n = 4) of patients had severe disease. An even distribution between endoscopic disease activity based on the Mayo endoscopic subscore was noted (18% remission, 34% mild, 31% moderate, 16% severe) (Table 1).

**Table 1:** Baseline demographic and clinical characteristics of both study cohorts.

	Cohort 1 (n = 108)	Cohort 2 (n = 28)
**General information**		
**Gender**		
*Female*	44 (42%)	12 (43%)
*Male*	60 (58%)	16 (57%)
**Age (years)**	45 [34, 57]	39 [25, 51]
**BMI**	25.6 [22.5–29.2]	23.3 [20.3–26.9]
**Smoking**		
*Yes*	57 (53%)	2 (7%)
* No*	51 (47%)	26 (93%)
**Laboratory parameters**		
CRP (mg/L)	2.4 [1.0, 5.5]	3.2 [0.7, 8.6]
Fecal calprotectin (µg/g)	134.5 [26.0, 684.5]	454.0 [139.0, 1325.0]
**Treatment**^**a**^		
5-ASA, topical	29 (27%)	NA
5-ASA, oral	73 (68%)	12 (43%)
Corticosteroids, topical	2 (2%)	NA
Corticosteroids, oral	8 (7%)	5 (18%)
Corticosteroids, iv	3 (3%)	NA
Immunosuppressant	18 (17%)	6 (21%)
Biologic agents	22 (20%)	14 (50%)
No treatment	12 (11%)	3 (11%)
**Clinical disease activity score**		
**Partial Mayo score**		
*Remission (< 2)*	54 (50%)	12 (48%)
*Mild (2–4)*	23 (21%)	5 (20%)
* Moderate (5–7)*	27 (25%)	6 (24%)
*Severe (> 7)*	4 (4%)	2 (8%)
**Mayo endoscopic subscore (MES)**		
*Remission*	11 (18%)	4 (20%)
*Mild*	21 (34%)	3 (15%)
*Moderate*	19 (31%)	3 (15%)
*Severe*	10 (16%)	10 (50%)
**Montreal classification**		
**Severity**		
* Mild (S1)*	NA	14 (56%)
* Moderate (S2)*	NA	10 (40%)
*Severe (S3)*	NA	1 (4%)
**Extent**		
*Proctitis (E1)*	33 (32%)	2 (8%)
* Distal (E2)*	45 (43%)	8 (32%)
*Pancolitis (E3)*	26 (25%)	15 (60%)

*Cohort 1:* Gender, age and BMI information missing for n = 4 patients (4%). Fecal calprotectin levels missing for n = 8 patients (7%). MES missing for n = 47 patients (44%). Montreal E classification missing for n = 4 patients (4%). *Cohort 2:* Fecal calprotectin levels missing for n = 2 patients (7%). Partial Mayo score missing for n = 3 patients (11%). MES missing for n = 8 patients (29%). Montreal E and Montreal S classifications missing for n = 3 patients (11%).

^a^Some patients received more than one treatment.

#### Cohort 2

The median patient age was 39 years [25, 51], with an even gender distribution (43% female vs. 57% male). A total of 11% of patients (n = 3) were on no form of treatment. According to the partial Mayo score, most patients were in remission (48%, n = 12), while 8% (n = 2) of patients had severe disease. According to the Mayo endoscopic subscore, 50% (n = 10) of patients had severe disease, while 20% (n = 4) were in remission. The majority of patients had pancolitis (Montreal E3), or 60% (n = 15) (Table 1).

### C4A3-HNE is negatively associated with disease activity in ulcerative colitis: *Cohort 1*

Correlation analysis was performed to examine the relationship between the biomarkers, disease scoring, established inflammatory markers, and common demographic covariates. A negative association between C4A3-HNE and disease activity was noted, where C4A3-HNE had a weak to moderate negative correlation to the Mayo endoscopic subscore (ρ = − 0.27, *p* < 0.05), the Mayo partial score, and the SCCAI score (both ρ = − 0.16). However, C4M showed no correlation with the Mayo endoscopic subscore, the Mayo partial score, or the SCCAI score. When combining C4A3-HNE and C4M into a ratio, a moderate negative correlation was observed with the Mayo endoscopic score (ρ = − 0.31, *p* < 0.05), CRP (ρ = − 0.34, *p* < 0.001), and FC (ρ = − 0.29, *p* < 0.01). C4M exhibited a strong positive correlation to CRP (ρ = 0.49, *p* < 0.0001), while C4A3-HNE showed no correlation. C4M showed a weak positive correlation to FC (ρ = 0.25, *p* < 0.05), while C4A3-HNE exhibited a weak negative correlation (ρ = − 0.13). When looking at demographic factors, no strong correlations were observed between age, gender, BMI, and smoking. C4M showed a weak correlation to age (ρ = 0.15) and gender (ρ = − 10), while C4A3-HNE exhibited a weak correlation to BMI (ρ = 0.12) (all *p* > 0.05) (Supplementary Table S3).

### C4A3-HNE and C4M reflect two different stages of mucosal damage in ulcerative colitis: *Cohort 1*

**Figure 4:**
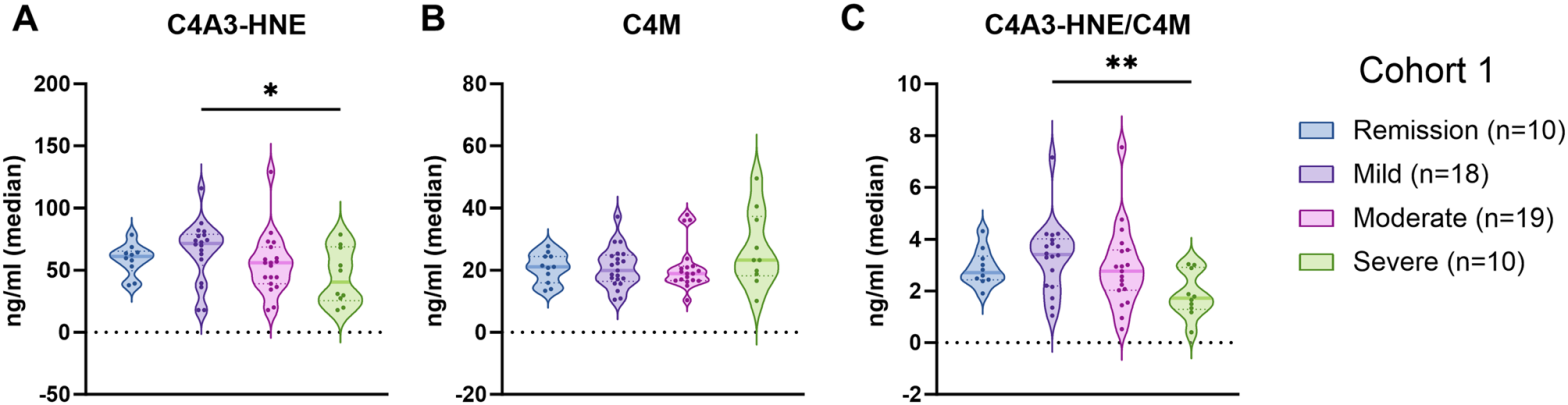
C4A3-HNE and C4M display contrasting patterns when subgrouped according to endoscopic disease activity**.** (**A**) C4A3-HNE is significantly elevated in patients with UC having mild endoscopic disease compared to patients with severe endoscopic disease (*p* < 0.05). (**B**) No significant difference in C4M levels was observed between groups. (**C**) The ratio of C4A3-HNE/C4M provides a more apparent separation, where patients with mild endoscopic disease have a significantly higher ratio between C4A3-HNE and C4M than patients with severe endoscopic disease (*p* < 0.01). Data is presented with violin plots, depicting the distribution and density of the data points with the horizontal line representing the median. Differences between groups were analysed using Dunn’s test with Bonferroni correction for MCP. *p < 0.05; **p < 0.01.

Patients were subgrouped according to the Mayo endoscopic subscore. C4A3-HNE was significantly elevated in patients with UC having mild endoscopic disease compared to patients with severe endoscopic disease (ng/ml [IQR]: 71.8 [59.9, 78.3] vs. 40.5 [28.1, 64.8], *p* < 0.05). Moreover, a numerical dose-dependent decrease was observed in the biomarker levels from patients with mild to severe disease (Fig. 4A, Supplementary Table S4). C4A3-HNE could significantly discriminate between patients with mild and severe endoscopic disease (AUC [95% CI]: 0.74 [0.55–0.94]) (Supplementary Table S2). When combined into a ratio with C4M, this separation became even more visible, where patients with mild endoscopic disease had a significantly higher C4A3-HNE/C4M ratio compared to patients with severe disease (ratio [IQR]: 3.4 [2.4, 3.9] vs. 1.6 [1.4, 2.6], *p* < 0.01) (Fig. 4B, Supplementary Table S4). C4A3-HNE/C4M provided better discrimination between the two groups than C4A3-HNE alone (AUC [95% CI]: 0.83 [0.67–0.98]) (Supplementary Table S2). C4M did not significantly differ between groups; however, it was numerically elevated in patients with severe endoscopic disease compared to remission, mild and moderate disease (ng/ml [IQR]: 23.3 [19.1, 34.1] vs. 21.1 [17.4, 24.5] vs. 20.0 [17.2, 24.6] vs. 19.0 [16.8, 21.2]) (Fig. 4C, Supplementary Table S4). To address the potential confounding effects of demographic variables, the data was adjusted for age, gender, BMI, and smoking (Supplementary Table S5). Additionally, the impact of treatment on biomarker levels was investigated. Patients on corticosteroids (iv) had higher levels of C4M and a lower ratio of C4A3-HNE/C4M compared to patients without the treatment (C4M ng/ml [IQR]: 38.0 [30.7, 39.2] vs. 20.0 [17.0, 24.5], *p* < 0.05; C4A3-HNE/C4M [IQR]: 1.3 [0.9, 1.8] vs. 2.9 [2.1, 3.8], *p* < 0.05) (Supplementary Table S8). Additionally, to assess the potential influence of steroid use on biomarker levels within endoscopic disease subgroups, steroid-free patients were analyzed separately (Supplementary Table S11).

### Inflammatory markers are lower in patients with high C4A3-HNE: *Cohort 1*

Patients were divided into two groups based on the median level of each biomarker (C4A3-HNE [62.7 ng/ml], C4M [20.2 ng/ml], C4A3-HNE/C4M [2.8]). Fecal calprotectin (FC) and C-reactive protein (CRP) levels were assessed between the groups. Results showed that patients with low levels of C4A3-HNE had numerically higher levels of both CRP and FC compared to patients with high biomarker levels (CRP (ml/L): 2.7 vs. 2.5–FC (µg/g): 193.0 vs. 140.0). Patients with low C4M levels had significantly lower levels of both CRP and FC compared to patients with high C4M levels (CRP (ml/L): 1.7 vs. 5.2–FC (µg/g): 83.5 vs. 236.0, *p* < 0.0001 and  < 0.05). Patients with a higher ratio between C4A3-HNE and C4M had significantly lower levels of both CRP and FC (CRP (ml/L): 4.3 vs. 2.0–FC (µg/g): 243.0 vs. 76.0, *p* < 0.01 and  < 0.05) (Supplementary Table S6).

### Parallel patterns: C4A4-HNE in *Cohort 2*

**Figure 5:**
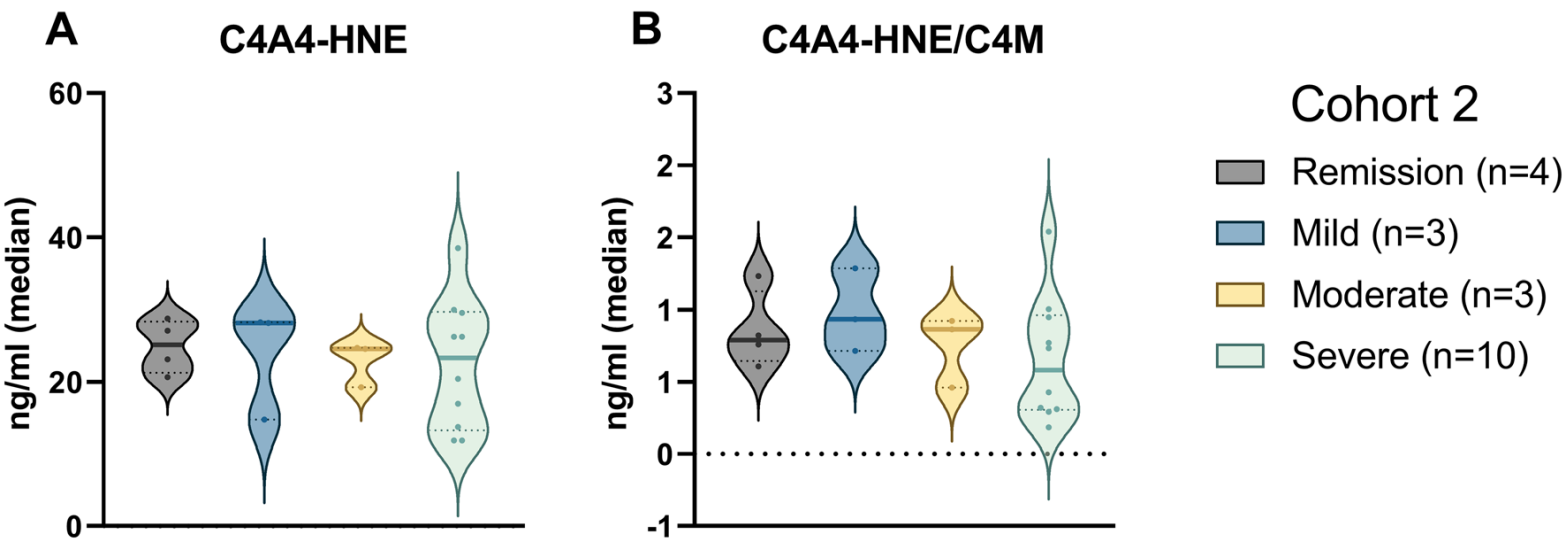
C4A4-HNE and C4A4-HNE/C4M are higher in patients with mild endoscopic ulcerative colitis. (**A**) C4A4-HNE showed a trend towards a dose-dependent decrease of biomarker levels from patients with mild endoscopic disease to patients with severe endoscopic disease. (**B**) The ratio between C4A4-HNE and C4M was lowest in patients with severe endoscopic disease. Data is presented with violin plots, depicting the distribution and density of the data points with the horizontal line representing the median.

Correlation analysis was performed to examine the relationship between the biomarkers, disease scoring, established inflammatory markers, and common demographic covariates. A weak negative correlation (ρ = − 0.13) was observed between the Mayo endoscopic subscore and C4A4-HNE, while C4M was previously shown to correlate positively with the modified Mayo endoscopic score (mMES)^19^. C4A4-HNE was otherwise positively correlated with the Mayo partial score (ρ = 0.22), CRP (ρ = 0.27), and FC (ρ = 0.39). The C4A4-HNE/C4M ratio displayed mild to moderate negative correlations to CRP (ρ = − 0.32), and the Mayo endoscopic subscore (ρ = − 0.28) C4A4-HNE exhibited a moderate positive correlation with gender (ρ = 0.38, *p* < 0.05). Additionally, a positive correlation was noted between both biomarkers and smoking (Supplementary Table S3). Patients in Cohort 2 with a recorded Mayo endoscopic subscore were grouped accordingly. C4A4-HNE showed a trend towards patients with mild disease having higher levels of the biomarker, with a dose-dependent decrease from patients with mild to severe endoscopic disease (ng/ml [IQR]: 28.2 [21.5, 28.2] vs. 24.6 [21.9, 24.7] vs. 23.3 [14.6, 28.8]) (Fig. 5A, Supplementary Table S4). The ratio between C4A4-HNE and C4M was numerically higher in patients with mild endoscopic disease compared to patients with severe endoscopic disease (ratio [IQR]: 0.9 [0.8, 1.1] vs. 0.6 [0.3, 0.9]) (AUC [95% CI]: 0.70 [0.38–1.00]) (Fig. 5B, Supplementary Table S4, Supplementary Table S2). To address the potential confounding effects of demographic variables, the data was adjusted for age, gender, and BMI (Supplementary Table S5). Furthermore, the impact of treatment on biomarker levels was investigated (Supplementary Table S9).

## Discussion

In the present study, we aimed to identify potential biomarkers of initial inflammatory insult in IBD. We successfully developed and validated two novel biomarker assays targeting specific neutrophil-derived type IV collagen fragments from the α3 and α4 chains. Quantification of these fragments in the serum of IBD patients revealed elevated levels of C4A3-HNE and C4A4-HNE compared to healthy donors. Of particular notice, the biomarkers were elevated in patients with UC having mild endoscopic disease, suggesting an association with the onset of inflammation and potentially early mucosal damage in IBD.

During the initial analysis of Cohort 1, we saw that C4A3-HNE was negatively correlated with the Mayo endoscopic subscore, the Simple Clinical Colitis Activity Index (SCCAI), the partial Mayo score, and fecal calprotectin. This came as a surprise since biomarkers of inflammation usually increase with disease activity^20^. To put these findings into perspective, C4M can be taken as an example. C4M is a neo-epitope of MMP-2, -9, and -12 mediated degradation of the α1 chain of type IV collagen and has been studied as a biomarker in IBD where it mainly reflects a severely disrupted intestinal barrier, mucosal damage, endoscopic disease severity, and positively associates with non-response to biologics^18,21–25^. MMP-2 and -9 are gelatinases primarily expressed by fibroblasts, activated monocytes, neutrophils, and T-cells^26,27^. MMP-12, also known as macrophage elastase, indirectly causes degradation of the intestinal epithelium as it is necessary for the migration of macrophages^28^. In the current study, C4M had a strong positive correlation to CRP and fecal calprotectin, supporting the notion that it reflects disease activity. When stratifying patients with UC according to their Mayo endoscopic subscore, C4A3-HNE was elevated in patients with mild endoscopic disease compared to those with severe endoscopic disease, and the biomarker levels decreased dose-dependently across the groups. In contrast, C4M levels were higher in patients with severe endoscopic disease, seemingly due to its association with disease activity. However, when combining the two markers into a ratio, the separation between patients with mild and severe endoscopic disease becomes even greater. By reporting a ratio of these two markers, we aimed to further explore their contrasting patterns. Patients with mild endoscopic disease have a higher ratio between C4A3-HNE and C4M, meaning that they have higher levels of C4A3-HNE than of C4M, and vice versa for patients with severe endoscopic disease.

**Figure 6:**
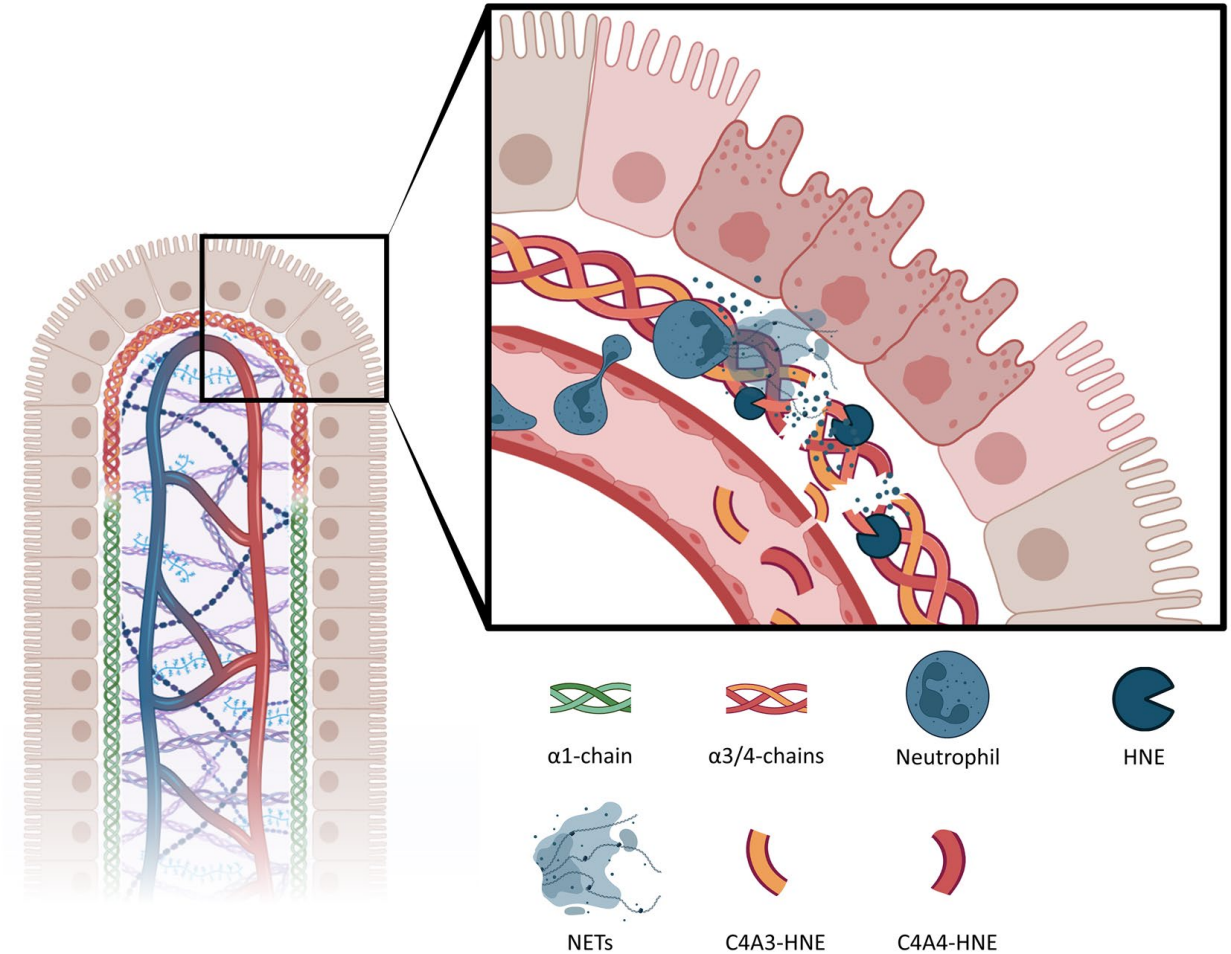
A schematic figure showcasing the generation of C4A3-HNE and C4A4-HNE in vivo. Activated neutrophils get recruited to the site of initial inflammation, migrating across the endothelium and through the ECM. Upon arrival the cells undergo NETosis, releasing cytotoxic proteins and proteases such as HNE. With the *α*3 and *α*4 chains of type IV collagen being solely expressed on the surface tip of the mucosa, they are easily compromised upon initial insult. Fragments of these neutrophil-degraded chains are released into circulation and can be measured in serum as C4A3-HNE and C4A4-HNE. In later and more severe stages of mucosal damage, the *α*1 chain is likewise degraded and released into circulation. *HNE* Human Neutrophil Elastase, *NETs* Neutrophil Extracellular Traps.

To interpret these results, the distribution of the alpha chains of type IV collagen within the iBM must be considered. Immunofluorescent staining of type IV collagen in the human colon has revealed that the α1 chain can be found throughout the iBM, from the crypts to the villi, and around smooth muscle cells in the muscularis mucosa. In contrast, the α3 and α4 chains were limited to the surface tip of the mucosa^14,15^. Furthermore, studies have shown that the infiltration of neutrophils is associated with tissue damage at mucosal surfaces, increasing barrier permeability^29^. Another way to think about these two different biomarkers is that C4A3-HNE could potentially reflect subclinical inflammation since it is easily degradable upon initial insult, being the uppermost chain of the iBM, and C4M would then reflect deeper ulceration when the damage has become clinically apparent (Fig. 6). These results emphasize the importance of investigating post-translationally modified protein fragments rather than solely focusing on the intact, full-length protein. Focusing on specific epitopes gives a more dynamic insight into the molecular mechanisms of the disease pathology. It should be noted that patients in endoscopic remission have C4A3-HNE levels on par with patients with severe endoscopic disease. A potential explanation for this could be that during remission, no injury or insult leading to inflammation should be present, meaning reduced recruitment and activation of neutrophils. In contrast, during severe endoscopic disease where large ulcerations have formed, the outermost layer of the mucosa has already been destroyed, and with it, the α3-chain.

In Cohort 2, C4A4-HNE showed a similar trend as C4A3-HNE, where patients with mild endoscopic ulcerative colitis had elevated levels compared to patients with severe endoscopic disease. In this same cohort, Domislovic et al.^19^ have previously shown C4M to be elevated in patients with moderate to severe ulcerative colitis compared to patients with mild endoscopic disease, suggesting an increase with inflammatory activity. Combining C4A4-HNE and C4M into a ratio gave similar results as C4A3-HNE/C4M. These results indicate that the two markers (C4A3-HNE and C4A4-HNE) behave similarly, which is to be expected based on their co-localization within the colonic mucosa. C4A4-HNE was generally less negatively correlated with disease activity than C4A3-HNE, although it negatively correlated with the Mayo endoscopic subscore. However, it is important to note that when performing any statistical analysis on small sample sizes, the parameters obtained are more easily influenced by outliers, missing values, and other fluctuations within the data. Therefore, it can be difficult to conclude the validity of these analyses.

Another methodological consideration is that most patients in both cohorts had received treatment at the time of sampling. When comparing biomarker levels between treated and non-treated patients, generally, no differences were observed, suggesting that treatment did not substantially affect the overall findings. Of note, the three patients in Cohort 1 using corticosteroids (iv) demonstrated significantly higher levels of C4M and lower of C4A3-HNE/C4M. A numerical difference was noted in C4A3-HNE levels, with patients using steroids having lower levels of the marker. This may speak into the overall hypothesis of C4M reflecting a more severe disease state than C4A3-HNE, since patients experiencing disease flares regularly receive corticosteroids prior to biologics^30^. However, we decided not to adjust for this covariate due to the limited number of patients. When patients using corticosteroids (iv, oral, topical) were collectively analyzed, no significant differences were noted in the biomarker levels, but a similar trend was observed in C4A3-HNE levels. Furthermore, excluding patients receiving corticosteroids did not alter the biomarker levels when stratified according to endoscopic disease severity. Regarding the potential confounding effects of demographic variables on the biomarker levels, a linear regression model was used to adjust for age, gender, BMI, and smoking, despite overall weak correlations with the biomarkers. For Cohort 2, smoking was significantly correlated with the biomarkers, but due to a sample size of only two smokers, and these patients not having a recorded endoscopic subscore, we decided not to adjust for this covariate. The overall trend for Cohort 1 stayed the same between patients grouped by the Mayo endoscopic subscore, whereas C4A4-HNE levels within Cohort 2 were affected. However, the assumption of normality of errors after modeling was not achieved due to heteroscedasticity, limiting the interpretability of these results. Furthermore, logistic regression analysis did not find a significant effect of age, gender, BMI, or smoking on the odds of having severe endoscopic disease (mild vs. severe). Of note, no patient information was available on concurrent autoimmune diseases for Cohort 1, and therefore their influence on biomarker levels cannot be definitively excluded. For future perspectives, a large and well-characterized clinical cohort would enable a thorough investigation of these markers. A direct comparison between C4A3-HNE and C4A4-HNE within the same patient population would provide further insight into which pathological processes they reflect. Long-term follow-up data on disease activity would also be useful for investigating whether these markers have predictive value regarding disease relapse. Furthermore, assessing these biomarkers in patients undergoing biological therapy would be valuable, as it may reveal distinct biomarker profiles that differ from those observed in the current study.

A more technical consideration regarding the proof-of-concept cleavage experiment is the small signal that can be noted for the peptide controls and the MMP-digested sample for C4A3-HNE. Firstly, the cleavage buffer gave a high background signal potentially due to its effects on the coater-antibody binding kinetics, leading to the values shown being background subtracted—therefore, residual buffer effects could produce an unaccounted-for signal. Secondly, the peptides might be unstable at the digestion temperature (37 °C) for long periods of time, and therefore some fragments could be passively released. Unspecific antibody binding can be ruled out with high certainty, as we have shown that the antibodies only bind their intended neo-epitopes—not the truncated or elongated fragments of interest, or the native type IV collagen protein. While the exact explanation remains elusive, we still repeatedly observe that HNE more frequently releases the neo-epitopes of interest. On this note, it can be observed that the C4A3-HNE fragment is detected in lower amounts compared with C4A4-HNE. Type IV collagen is known to be difficult to cleave due to the multiple non-collagenous interruptions present within the helical region, as corroborated by the inability of HNE to release the neo-epitopes after incubation with native type IV collagen. Based on this, we can assume that type IV collagen needs significant pre-processing with other proteases before HNE can fully access its cleavage site. Predicting this pre-processing cascade is challenging due to the highly dynamic microenvironment present in vivo. Therefore, the low production of the C4A3 fragment could be attributed to insufficient pre-processing when working with the protein in vitro, resulting in suboptimal HNE binding affinity or ability. On the other hand, there could also be differences in the pathological processes or cellular pathways leading up to the generation of the two different fragments. This requires a much deeper investigation than this paper provides and could be a topic for future perspectives, by applying and investigating i.e., an in vitro neutrophil cell model that naturally produces the fragments upon relevant stimuli.

This study demonstrates the biological and clinical relevance of two novel biomarkers reflecting neutrophil-mediated degradation of the α3 and α4 chains of type IV collagen, C4A3-HNE and C4A4-HNE. A negative association was observed between the two biomarkers and disease activity, where patients with mild endoscopic ulcerative colitis had elevated levels compared to patients with severe endoscopic disease. Furthermore, combining the markers with an MMP-derived fragment from the α1 chain of type IV collagen provided information on two distinct stages of mucosal damage, demonstrating that the epitope matters. These biomarkers could provide early detection of mucosal damage and may be used to monitor disease relapse in patients in remission, or as an indicator of when endoscopic assessment is needed, reducing the need for frequent invasive procedures.

### Supplementary Information

Supplementary Information.

## Data Availability

The datasets generated during and/or analysed during the current study are available from the corresponding author on reasonable request.
