# Supplementary material for: Neutrophil-mediated type IV collagen degradation is elevated in patients with mild endoscopic ulcerative colitis reflecting early mucosal destruction
Source: Sci Rep. 2024 Jan 18;14:1641. doi: 10.1038/s41598-024-52208-y (PMC10796406; doi:10.1038/s41598-024-52208-y)
Supplement: Supplementary file 1 — Supplementary Information. [file 41598_2024_52208_MOESM1_ESM.docx]

Supplementary

***
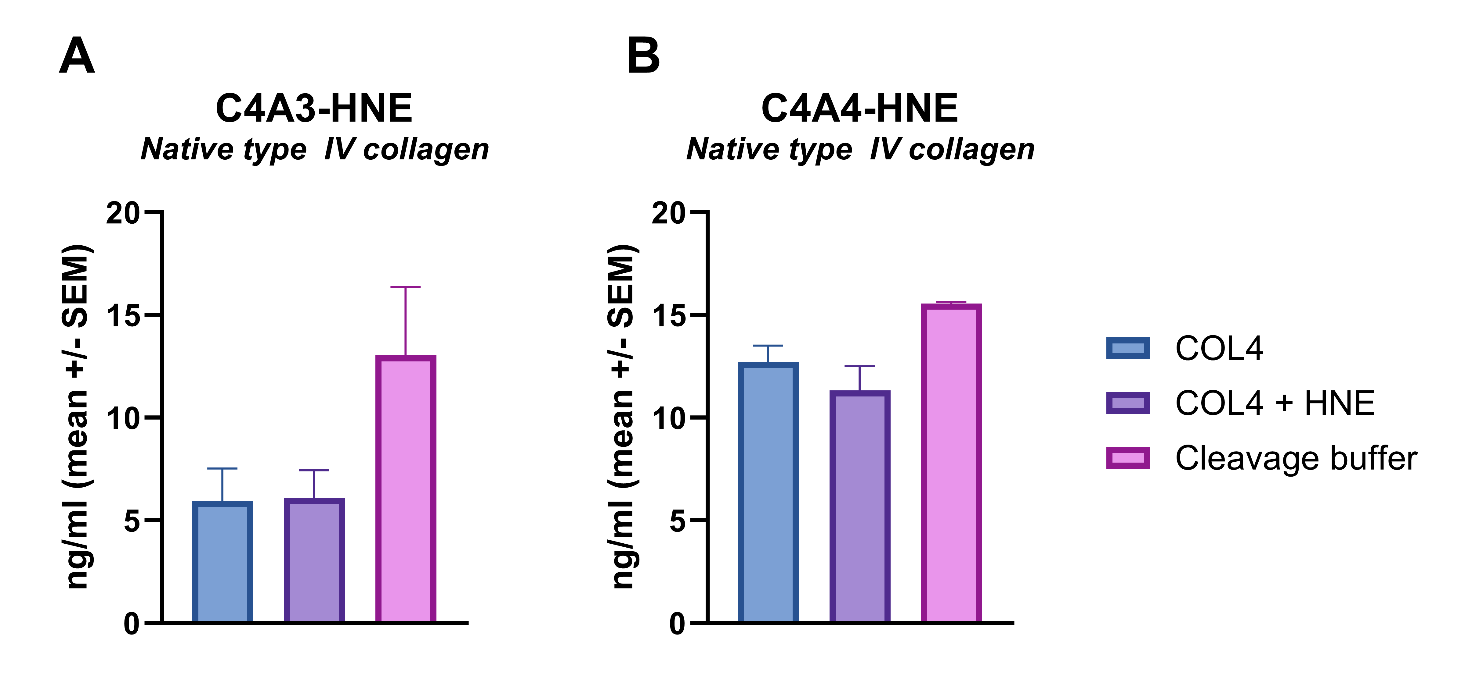
***

**Figure S1**. Native type IV collagen was incubated with active HNE for 4 hours. The intact protein (without protease) and cleavage buffer were used as negative controls. HNE was not able to release the C4A3-HNE **(A)** or C4A4-HNE **(B)** fragment from the native protein upon incubation. Cleavages were carried out in two biological replicates, with two technical replicates within each experiment.

**Table S1.** Technical validation parameters of C4A3-HNE and C4A4-HNE.

| **PARAMETER** | **C4A3-HNE** | **C4A4-HNE** |
| --- | --- | --- |
| **Measurement range in serum (LLOQ-ULOQ)** | 15.6 – 1000.0 (ng/ml) | 5.62 – 444.44 (ng/ml) |
| **Lower limit of blank (LLOB)** | 0.56 ng/ml | 1.36 ng/ml |
| **Mean IC50** | 73.12 ng/ml | 33.55 ng/ml |
| **Mean slope** | 1.01 | 0.95 |
| **Spiking recovery of serum in serum** | -4% – 22% | -2.7 – 14.7% |
| **Intra-assay variation CV%** | ≤ 13% | ≤ 7% |
| **Inter-assay variation CV%** | ≤ 9% | ≤ 12% |
| **Dilution recovery (1+3)** | 88 – 122% | 80 – 109% |
| **Freeze/thaw recovery (5 cycles)** | 77 – 114% | 86 – 105% |
| **Biotin recovery, low/high^a,b^** | 98%/82% | 112%/82% |
| **Lipemia recovery, low/high^c^** | 108%/103% | 115%/117% |
| **Hemoglobin recovery, low/high^d^** | 104%/88.9% | 112%/95% |

^a^C4A3: 5/80 ng/ml, ^b^C4A4: 5/100 ng/ml, ^c^1.5/5 mg/ml, ^d^2.5/5 mg/ml

**Table S2.** AUC values for Receiver Operating Characteristic (ROC) analysis of the biomarkers distinguishing between diseased and healthy, and mild vs. severe endoscopic disease activity. Biomarkers with AUCs over 0.70 were considered to have acceptable discriminative capabilities.

| **Comparison** | **Biomarker** | **AUC [95 % CI]** | **Sensitivity (%)** | **Specificity (%)** | ***p*** |
| --- | --- | --- | --- | --- | --- |
| **Proteogenex** | | | | | |
| **HD vs. CD** | **C4A3-HNE** | **0.82 [0.67-0.92]** | **79** | **88** | **<0.001** |
| **HD vs. UC** |  | **0.89 [0.76-0.96]** | **75** | **100** | **<0.0001** |
| **HD vs. CD** | **C4A4-HNE** | **0.91 [0.70-0.99]** | **90** | **100** | **0.002** |
| **HD vs. UC** |  | **1.00 [0.87-1.00]** | **100** | **100** | **<0.0001** |
| **Cohort 1** | | | | | |
| **HD vs. CD** | **C4A3-HNE** | **0.76 [0.65-0.84]** | **58** | **94** | **<0.0001** |
| **HD vs. UC** |  | **0.74 [0.66-0.82]** | **58** | **97** | **<0.0001** |
| **Mild vs. severe** | **C4A3-HNE** | **0.74 [0.55-0.94]** | **70** | **78** | **0.035** |
| Mild vs. severe | C4M | 0.63 [0.4-0.87] | 80 | 48 | 0.298 |
| **Mild vs. severe** | **C4A3-HNE/C4M** | **0.83 [0.67-0.98]** | **100** | **67** | **<0.01** |
| **Cohort 2** | | | | | |
| **HD vs. CD** | **C4A4-HNE** | **0.79 [0.71-0.86]** | **69.4** | **90.6** | **<0.0001** |
| **HD vs. UC** |  | **0.76 [0.63-0.89]** | **63.2** | **90.6** | **<0.001** |
| Mild vs. severe | C4A4-HNE | 0.57 [0.20-0.93] | 70 | 67 | 0.735 |
| Mild vs. severe | C4A4-HNE/C4M | 0.70 [0.38-1.00] | 50 | 100 | 0.311 |

**Table S3.** Correlation analysis was performed to investigate the relationship between biomarkers, disease scoring, established inflammatory markers, and common demographic covariates. Spearman correlation coefficients are presented with their corresponding p-values for each cohort. P-values <0.05 were considered significant.

|  | Cohort 1 | | |  | Cohort 2 | |
| --- | --- | --- | --- | --- | --- | --- |
|  | **C4A3-HNE** | **C4M** | **C4A3-HNE/C4M** |  | **C4A4-HNE** | **C4A4-HNE/C4M** |
| **Mayo partial** | -0.16  (0.113) | 0.03  (0.730) | -0.14  (0.170) |  | 0.22  (0.300) | 0.10  (0.648) |
| **Mayo endoscopic** | **-0.27  (0.039)** | 0.06  (0.624) | **-0.31  (0.020)** |  | -0.13  (0.577) | -0.28  (0.227) |
| **SCCAI** | -0.16  (0.111) | 0.06  (0.554) | -0.19  (0.056) |  | *NA* | *NA* |
| **CRP** | 0.01  (0.950) | **0.49  (<0000.1)** | **-0.34 (<000.1)** |  | 0.27  (0.167) | -0.32  (0.101) |
| **Fecal Calprotectin** | -0.13  (0.210) | **0.25  (0.013)** | **-0.29  (0.005)** |  | 0.39  (0.051) | -0.10  (0.611) |
| **Age** | 0.09  (0.363) | 0.15  (0.143) | -0.01  (0.905) |  | 0.30  (0.121) | 0  (0.995) |
| **Gender** | 0.02  (0.811) | -0.10  (0.294) | 0.02  (0.839) |  | **0.38  (0.044)** | 0.23  (0.234) |
| **BMI** | 0.12  (0.246) | 0.06  (0.576) | 0.02  (0.861) |  | 0.08  (0.690) | 0.18  (0.353) |
| **Smoking** | 0.04 (0.699) | -0.05 (0.611) | 0.05 (0.642) |  | **0.47 (0.011)** | **0.43 (0.021)** |

**Table S4.** Biomarker levels at baseline between patients grouped by the Mayo endoscopic subscore. Values are presented as median [IQR]. P-values are Bonferroni-corrected for MCP, and p-values <0.05 were considered significant.

|  |  |  |  |  | *p*-unadjusted | | | | | *p*-adjusted | | | |
| --- | --- | --- | --- | --- | --- | --- | --- | --- | --- | --- | --- | --- | --- |
| **Biomarker** | **Remission (0)** | **Mild (1)** | **Moderate (2)** | **Severe (3)** | **0 vs. 1** | **1 vs. 2** | | **1 vs. 3** | | **0 vs. 1** | | **1 vs. 2** | **1 vs. 3** |
| **Cohort 1** | | | | | | | | | | | | | |
| C4A3-HNE | 61.2 [54.4 - 64.3] | 71.8 [59.9 - 78.3] | 56.1 [41.8 - 64.3] | 40.5 [28.1 - 64.8] | 0.269 | | 0.044 | | **0.012** | | 0.807 | 0.131 | **0.035** |
| C4M | 21.1 [17.4 - 24.5] | 20.0 [17.2 - 24.6] | 19.0 [16.8 - 21.2] | 23.3 [19.1 - 34.1] | 0.832 | | 0.696 | | 0.249 | | 1.000 | 1.000 | 0.748 |
| C4A3-HNE/C4M | 2.7 [2.5 -  3.2] | 3.4 [2.4 - 3.9] | 2.8 [2.1 -  3.6] | 1.6 [1.4 -  2.6] | 0.440 | | 0.219 | | **0.003** | | 1.000 | 0.656 | **0.008** |
| **Cohort 2** | | | | | | | | | | | | | |
| C4A4-HNE | 25.1 [22.5 - 27.5] | 28.2 [21.5 - 28.2] | 24.6 [21.9 - 24.7] | 23.3 [14.6 - 28.8] | 0.941 | | 0.581 | | 0.669 | | 1.000 | 1.000 | 1.000 |
| C4A4-HNE/C4M | 0.8 [0.7 -  0.9] | 0.9 [0.8 - 1.1] | 0.9 [0.7 -  0.9] | 0.6 [0.3 -  0.9] | 0.619 | | 0.535 | | 0.182 | | 1.000 | 1.000 | 0.545 |

**Table S5.** Biomarker levels at baseline between patients grouped by the Mayo endoscopic subscore adjusted for age, gender and BMI using linear regression. Cohort 1 is additionally adjusted for smoking. Values are presented as estimated means [95% CI]. Adjusted p-values are Bonferroni corrected for MCP. *P*-values <0.05 were considered significant.

|  |  |  |  |  | *p*-unadjusted | | | *p*-adjusted | | |
| --- | --- | --- | --- | --- | --- | --- | --- | --- | --- | --- |
| **Biomarker** | **Remission (0)** | **Mild (1)** | **Moderate (2)** | **Severe (3)** | **0 vs. 1** | **1 vs. 2** | **1 vs. 3** | **0 vs. 1** | **1 vs. 2** | **1 vs. 3** |
| **Cohort 1** | | | | | | | | | | |
| C4A3-HNE | 56.9 [47.3 - 68.5] | 58.7 [44.6 - 77.2] | 50.9 [40.1 - 64.7] | 40.2 [26.8 - 60.1] | 0.852 | 0.469 | 0.131 | 1.000 | 0.912 | 0.394 |
| C4M | 19.9 [16.6 - 24.0] | 19.9 [17.0 - 23.2] | 19.8 [16.7 - 23.4] | 24.1 [17.7 - 32.8] | 0.982 | 0.977 | 0.263 | 1.000 | 1.000 | 0.789 |
| C4A3-HNE/C4M | 2.8 [2.2 -  3.6] | 2.9 [2.2 - 3.8] | 2.6 [1.9  - 3.5] | 1.7 [1.1 -  2.5] | 0.923 | 0.599 | **0.020** | 1.000 | 1.000 | 0.059 |
| **Cohort 2** | | | | | | | | | | |
| C4A4-HNE | 28.6 [15.59 - 52.6] | 25.8 [13.54 - 49.0] | 33.2 [16.98 - 65.1] | 25.7 [16.42 - 40.2] | 0.725 | 0.468 | 0.994 | 1.000 | 1.000 | 1.000 |
| C4A4-HNE/C4M | 1.1 [0.37  - 3.1] | 1.3 [0.42 - 3.9] | 1.2 [0.37 - 3.8] | 0.7 [0.33 - 1.6] | 0.731 | 0.906 | 0.192 | 1.000 | 1.000 | 0.576 |

**Table S6.** CRP and fecal calprotectin (FC) levels at baseline for patients grouped by median C4A3-HNE, C4M, and C4A3-HNE levels within Cohort 1. Values are presented as median [IQR]. Differences between groups were analysed using Mann-Whitney U-test.

|  | C4A3-HNE | | | C4M | | | C4A3-HNE/C4M | | |
| --- | --- | --- | --- | --- | --- | --- | --- | --- | --- |
|  | **Low** | **High** | ***p*** | **Low** | **High** | ***p*** | **Low** | **High** | ***p*** |
| **CRP (ml/L)** | 2.7  [1.0, 5.9] | 2.5  [1.0, 5.4] | 0.878 | 1.7  [1.0, 2.8] | 5.2  [1.8, 11.0] | **<0.0001** | 4.3  [1.2, 12.5] | 2.0  [1.0, 3.7] | **0.004** |
| **FC (µg/g)** | 193.0  [42.0, 801.0] | 140.0  [23.0, 601.0] | 0.168 | 83.5  [21.5, 347.8] | 236.0  [37.2, 1119.8] | **0.042** | 243.0  [43.5, 1076.8] | 76.0  [23.0, 378.0] | **0.022** |

**Table S7.** Multivariate logistic regression examining the association between biomarkers, common demographic factors, and the odds of having severe endoscopic disease. The binary outcome variable used in the model was (0) vs. severe (1) disease. Demographic factors not associated with odds of having severe endoscopic disease. P-values <0.05 were considered significant.

| Cohort 1 | | | | | | | | |
| --- | --- | --- | --- | --- | --- | --- | --- | --- |
|  | **C4A3-HNE** | |  | **C4M** | |  | **C4A3-HNE/C4M** | |
| **Parameter** | **Estimate** | **p** |  | **Estimate** | **p** |  | **Estimate** | **p** |
| Biomarker | -1.456 | 0.090 |  | 1.634 | 0.201 |  | **-3.063** | **0.034** |
| Age | 0.022 | 0.540 |  | -0.010 | 0.762 |  | 0.051 | 0.266 |
| Gender | 0.673 | 0.481 |  | 0.602 | 0.511 |  | 1.278 | 0.255 |
| BMI | -0.093 | 0.383 |  | -0.061 | 0.496 |  | -0.105 | 0.387 |
| Smoking | -0.866 | 0.288 |  | -0.170 | 0.810 |  | -1.468 | 0.152 |
| **Cohort 2** | | | | | | | | |
|  | **C4A4-HNE** | |  | **C4A4-HNE/C4M** | |  |  | |
| **Parameter** | **Estimate** | **p** |  | **Estimate** | **p** |  |  |  |
| Biomarker | 13.763 | 0.532 |  | -8.708 | 0.274 |  |  |  |
| Age | -0.212 | 0.480 |  | -0.293 | 0.336 |  |  |  |
| Gender | -5.054 | 0.544 |  | -3.389 | 0.403 |  |  |  |
| BMI | 0.454 | 0.600 |  | 0.478 | 0.431 |  |  |  |

**Table S8.** Biomarker levels between patients in Cohort 1 that received some form of treatment and those who did not. Values are presented as median [IQR]. Differences between groups were analyzed using Mann-Whitney U-test. P-values <0.05 were considered significant.

|  | Corticosteroids, iv | |  | Corticosteroids, oral | |  | Corticosteroids, topical | |  | Corticosteroids, all | |  |
| --- | --- | --- | --- | --- | --- | --- | --- | --- | --- | --- | --- | --- |
| **Biomarker** | **Yes (n=3)** | **No (n=105)** | ***p*** | **Yes (n=8)** | **No (n=100)** | ***p*** | **Yes (n=2)** | **No (n=106)** | ***p*** | **Yes (n=13)** | **No (n=95)** | ***p*** |
| C4A3-HNE | 52.4 [36.2 - 53.2] | 63.2 [42.0 - 76.2] | 0.140 | 56.8 [43.2 - 71.3] | 62.8 [40.8 - 76.7] | 0.713 | 47.8 [39.4 - 56.1] | 62.8 [43.2 - 75.7] | 0.426 | 52.4 [39.4 - 64.5] | 63.2 [44.4 - 77.5] | 0.169 |
| C4M | 38.0 [30.7 - 39.2] | 20.0 [17.0 - 24.5] | **0.025** | 19.9 [16.2 - 23.7] | 20.4 [17.2 - 25.2] | 0.657 | 16.3 [15.0 - 17.5] | 20.6 [17.1 - 25.2] | 0.214 | 23.3 [16.6 - 23.8] | 20.2 [17.2 - 25.0] | 0.793 |
| C4A3-HNE/C4M | 1.3 [0.9 - 1.8] | 2.9 [2.1 - 3.8] | **0.031** | 3.0 [2.6 - 3.2] | 2.8 [2.0 - 3.8] | 0.875 | 3.2 [2.4 - 3.9] | 2.8 [2.1 - 3.7] | 0.865 | 2.8 [1.7 - 3.0] | 2.8 [2.1 - 3.8] | 0.369 |
|  | **Biologic agents** | |  | **Immunosuppressant** | |  | **5-ASA, oral** | |  | **5-ASA, topical** | |  |
| **Biomarker** | **Yes (n=22)** | **No (n=86)** | ***p*** | **Yes (n=18)** | **No (n=90)** | ***p*** | **Yes (n=73)** | **No (n=35)** | ***p*** | **Yes (n=29)** | **No (n=79)** | ***p*** |
| C4A3-HNE | 62.8 [41.1 - 80.7] | 62.8 [43.2 - 74.1] | 0.586 | 73.4 [52.2 - 77.4] | 62.7 [39.4 - 74.6] | 0.087 | 59.9 [39.4 - 73.0] | 66.3 [47.3 - 79.6] | 0.144 | 58.8 [38.5 - 72.6] | 62.8 [44.4 - 77.2] | 0.601 |
| C4M | 20.5 [17.6 - 29.0] | 20.0 [16.8 - 24.5] | 0.371 | 18.4 [17.1 - 20.2] | 21.0 [17.1 - 25.3] | 0.677 | 21.1 [17.3 - 25.2] | 19.5 [16.8 - 24.5] | 0.457 | 19.5 [16.3 - 23.3] | 20.8 [17.2 - 25.4] | 0.215 |
| C4A3-HNE/C4M | 2.7 [2.4 - 3.3] | 2.9 [1.9 - 3.8] | 0.736 | 3.6 [2.5 - 4.2] | 2.8 [1.9 - 3.7] | 0.129 | 2.6 [1.9 - 3.7] | 3.0 [2.7 - 4.2] | 0.055 | 3.0 [1.7 - 4.2] | 2.7 [2.1 - 3.7] | 0.448 |

**Table S9.** Biomarker levels between patients in Cohort 2 that received some form of treatment and those who did not. Values are presented as median [IQR]. Differences between groups were analyzed using Mann-Whitney U-test. P-values <0.05 were considered significant.

|  | Corticosteroids, oral | |  | Biologic agents | |  |
| --- | --- | --- | --- | --- | --- | --- |
| **Biomarker** | **Yes (n=5)** | **No (n=23)** | ***p*** | **Yes (n=14)** | **No (n=14)** | ***p*** |
| C4A4-HNE | 23.2 [19.2 - 28.5] | 26.2 [24.6 - 38.5] | 0.322 | 23.9 [15.9 - 28.8] | 25.4 [20.9 - 28.7] | 0.613 |
| C4A4-HNE/C4M | 0.8 [0.5 - 1.0] | 0.8 [0.3 - 0.9] | 0.569 | 0.7 [0.4 - 0.9] | 0.9 [0.6 - 1.0] | 0.448 |
|  | **Immunosuppressant** | |  | **5-ASA - oral** | |  |
| **Biomarker** | **Yes (n=6)** | **No (n=22)** | ***p*** | **Yes (n=12)** | **No (n=16)** | ***p*** |
| C4A4-HNE | 24.7 [19.5 - 28.9] | 24.4 [14.5 - 26.9] | 0.401 | 20.9 [13.3 - 28.4] | 25.4 [22.0 - 29.0] | 0.194 |
| C4A4-HNE/C4M | 0.7 [0.5 - 1.0] | 0.8 [0.5 - 0.9] | 0.845 | 0.7 [0.4 - 0.9] | 0.8 [0.6 - 1.0] | 0.286 |

**Table S10.** Biomarker levels at baseline between patients grouped by the Partial Mayo score. Values are presented as median [IQR]. P-values are Bonferroni-corrected for MCP, and p-values <0.05 were considered significant.

|  |  |  |  |  | *p*-unadjusted | | | *p*-adjusted | | | |
| --- | --- | --- | --- | --- | --- | --- | --- | --- | --- | --- | --- |
| **Biomarker** | **Remission (0)** | **Mild (1)** | **Moderate (2)** | **Severe (3)** | **0 vs. 1** | **1 vs. 2** | **1 vs. 3** | | **0 vs. 1** | **1 vs. 2** | **1 vs. 3** |
| **Cohort 1** | | | | | | | | | | | |
| C4A3-HNE | 66.2 [49.3, 78.7] | 60.3 [39.6, 68.7] | 58.1 [44.4, 76.2] | 29.3 [25.7, 41.5] | 0.178 | 0.701 | 0.195 | | 0.534 | 1.000 | 0.586 |
| C4M | 19.8 [17.2, 24.6] | 21.7 [18.5, 25.1] | 19.5 [16.6, 25.3] | 21.1 [18.2, 29.9] | 0.366 | 0.335 | 0.935 | | 1.000 | 1.000 | 1.000 |
| C4A3-HNE/C4M | 3.0 [2.3, 4.0] | 2.7 [1.8, 2.8] | 3.0 [2.1, 3.6] | 1.4 [1.0, 2.3] | 0.092 | 0.210 | 0.392 | | 0.276 | 0.630 | 1.000 |
| **Cohort 2** | | | | | | | | | | | |
| C4A4-HNE | 22.8 [18.1, 25.3] | 28.3 [24.6, 33.3] | 24.6 [19.5, 29.4] | 26.2 [26.2, 26.2] | 0.155 | 0.540 | 0.884 | | 0.466 | 1.000 | 1.000 |
| C4A4-HNE/C4M | 0.8 [0.6, 1.0] | 0.9 [0.3, 0.9] | 0.7 [0.5, 0.9] | 1.2 [1.0, 1.3] | 0.822 | 0.875 | 0.269 | | 1.000 | 1.000 | 0.808 |

**Table S11.** Biomarker levels at baseline between patients grouped by the Mayo endoscopic subscore, excluding patients on corticosteroids (iv, oral, topical). Values are presented as median [IQR]. P-values are Bonferroni-corrected for MCP, and p-values <0.05 were considered significant.

|  |  |  |  |  | *p*-unadjusted | | | *p*-adjusted | | | |
| --- | --- | --- | --- | --- | --- | --- | --- | --- | --- | --- | --- |
| **Biomarker** | **Remission (0)** | **Mild (1)** | **Moderate (2)** | **Severe (3)** | **0 vs. 1** | **1 vs. 2** | **1 vs. 3** | | **0 vs. 1** | **1 vs. 2** | **1 vs. 3** |
| **Cohort 1** | | | | | | | | | | | |
| C4A3-HNE | 62.6 [58.1 - 64.4] | 71.8 [59.9 - 78.3] | 56.2 [44.4 - 64.3] | 28.7 [21.8 - 58.8] | 0.346 | 0.087 | **0.028** | | 1 | 0.261 | 0.083 |
| C4M | 2.6 [2.4 -  3.3] | 3.4 [2.4 -  3.9] | 2.8 [2.2 -  3.6] | 1.5 [1.3 -  1.8] | 0.478 | 0.365 | **0.002** | | 1 | 1.000 | **0.007** |
| C4A3-HNE/C4M | 21.3 [19.3 - 24.5] | 20.0 [17.2 - 24.6] | 19.0 [17.0 - 20.8] | 25.3 [20.7 - 34.1] | 0.578 | 0.537 | 0.204 | | 1 | 1.000 | 0.613 |
